# Successful pregnancies and healthy live births using frozen-thawed sperm retrieved by a new modified Hotchkiss procedure in males with retrograde ejaculation: first case series

**DOI:** 10.1186/s12610-015-0021-4

**Published:** 2015-05-15

**Authors:** Melanie Philippon, Gilles Karsenty, Benjamin Bernuz, Blandine Courbiere, Thierry Brue, Jacqueline Saïas-Magnan, Jeanne Perrin

**Affiliations:** AP-HM, La Conception, Service d’Endocrinologie, Diabète et Maladies Métaboliques, Centre de Référence des Maladies Rares d’Origine Hypophysaire DEFHY, 13385 cedex 15 Marseille, France; Department of Urology, AP-HM La Conception, 147 bd Baille, 13385 Cedex 5 Marseille, France; Neuro rehabilitation Unit, Leon Berard Hospital, bd du Dr Armanet, Hyères, France; Pôle Femmes-Parents-Enfants, Centre clinico-biologique d’Assistance Médicale à la Procréation, AP-HM La Conception, 147 Bd Baille, 13 005 Marseille, France; Institut Méditerranéen de Biodiversité et d’Ecologie Marine et Continentale (IMBE), CNRS - IRD, Aix Marseille Université, Univ-Avignon, Biogénotoxicologie, Santé Humaine et Environnement, 27, Boulevard Jean-Moulin, F-13385 Cedex 05 Marseille, France; CECOS Laboratory Biology of Reproduction, Pôle Femmes-Parents-Enfants, 147 bd Baille, 13385 Cedex 5 Marseille, France; Aix Marseille Université, CNRS, CRN2M UMR 7286, 13344 cedex 15 Marseille, France

**Keywords:** Male infertility, Bladder, Spinal cord injury, Diabetes, Post-thaw evaluation, Infertilité masculine, Vessie, Lésion médullaire, Diabète, Test de décongélation

## Abstract

**Background:**

In couples presenting with retrograde ejaculation refractory to medical treatment, the first choice of fertility treatment should be Assisted Reproductive Techniques using rapidly purified spermatozoa retrieved from post-ejaculatory urine. The Hotchkiss technique and modified variants are simple and efficient for retrieving sperm from the bladder. We developed a new protocol, including a novel modified Hotchkiss technique involving sperm cryopreservation.

The aim was to study the pregnancy rate and birth rate achieved by intra cytoplasmic sperm injection (ICSI) using frozen-thawed sperm retrieved from the bladder with this novel modified Hotchkiss technique in patients with refractory retrograde ejaculation.

**Results:**

In this descriptive retrospective, single-center study, we analyzed the local database of all patients who banked sperm at the CECOS Laboratory Biology of Reproduction of La Conception University Hospital, Marseille, France, between 2004 and 2014.

A total of 2171 patients banked sperm during this time, including 63 patients with retrograde ejaculation, of whom ten patients banked sperm that had been retrieved by the modified Hotchkiss technique.

These ten couples underwent 26 ICSI cycles: nine clinical pregnancies were achieved in six couples, including eight after fresh embryo transfer and one after thawed embryo transfer, resulting in seven live births. The average live birth rate per transfer was 28 %.

**Conclusions:**

We report the largest series of births using frozen-thawed spermatozoa retrieved from post-ejaculatory urine by a modified Hotchkiss technique.

This series of births demonstrates that this new modified Hotchkiss technique allows for successful association with sperm cryopreservation, leading to an efficient and easy management of couples with refractory retrograde ejaculation.

## Résumé

**Contexte:**

Chez les couples dont l’homme présente une éjaculation rétrograde réfractaire au traitement médical, la première ligne de traitement de l’infertilité est le recours à l’aide médicale à la procréation (AMP) utilisant des spermatozoïdes purifiés des urines après éjaculation.

La technique de Hotchkiss et les techniques de Hotchkiss modifiées sont simples et efficaces pour récupérer les spermatozoïdes de la vessie. Nous avons développé un nouveau protocole avec une nouvelle technique de Hotchkiss modifiée impliquant une cryoconservation des spermatozoïdes obtenus.

L’objectif de cette étude était d’étudier les taux de grossesses et de naissances obtenues après injection intra cytoplasmique de spermatozoïdes (ICSI) provenant de paillettes congelées à partir des spermatozoïdes purifiés des urines avec la nouvelles méthode de Hotchkiss modifiée, chez les patients atteints d’éjaculation rétrograde réfractaire.

**Résultats:**

Dans cette étude descriptive monocentrique rétrospective, nous avons analysé la base de données locale de tous les patients ayant conservé du sperme au CECOS du laboratoire de biologie de la reproduction de l’hôpital universitaire de la Conception à Marseille, France, entre 2004 et 2014.

Un total de 2171 patients a conservé du sperme pendant cette période. Soixante trois présentaient une éjaculation rétrograde, parmi lesquels 10 ont conservé du sperme qui avait été purifié des urines selon la méthode de Hotchkiss modifiée.

Ces 10 couples ont réalisé 26 tentatives d’ICSI. 9 grossesses cliniques ont été obtenues chez 6 couples; 8 après transfert d’embryon frais, et une après transfert d’embryon congelé, ayant conduit à 7 naissances. Le taux moyen de naissance par transfert était de 28 %.

**Conclusions:**

Nous rapportons ici la plus large série de naissances utilisant des paillettes de spermatozoïdes purifiés des urines après éjaculation rétrograde selon une méthode de Hotchkiss modifiée.

Cette série de naissances démontre que cette nouvelle technique de Hotchkiss permet une association réussie avec la cryoconservation du sperme, permettant une prise en charge facile et efficace des couples avec éjaculation rétrograde réfractaire.

## Background

Retrograde ejaculation (RE) accounts for less than 2 % of cases of male infertility [1, 2]. This condition can occur as a result of spinal cord lesions, neuropathies (diabetic autonomic neuropathy and multiple sclerosis), retroperitoneal surgery, acquired anatomic aetiologies (bladder neck surgery and transurethral resection of the prostate), congenital abnormalities, or pharmacological treatments (psychotropic medications and a-adrenergic blockers), and it can also be idiopathic [2–4].

In couples presenting with retrograde ejaculation refractory to medical treatment, the first choice of fertility treatment should be assisted reproductive technology (ART) using rapidly purified spermatozoa retrieved from post-ejaculatory urine [4, 5]. However, due to its high osmolarity and low pH, urine is cytotoxic to spermatozoa [6–8]. For urinary sperm retrieval, the technique described by Hotchkiss *et al.* [9] is simple and efficient. Using this technique, the bladder is emptied and washed with Ringer’s lactate via catheterization, and then a small amount of the same solution is instilled into the bladder before removing the catheter. The patient is then instructed to ejaculate. Post-ejaculatory bladder contents are obtained by either voiding or catheterization, and the suspended spermatozoa are used for intrauterine insemination (IUI).

Several modified Hotchkiss techniques (MHT) have been described, which involve the instillation of additional types of culture media and/or the use of additional ARTs to increase sperm viability, motility, and pregnancy and birth rates [4, 10]. According to a systematic review by Jefferys *et al.* [4] based on 13 clinical cases [11–18], 8 live births have been accomplished by IUI using fresh sperm. One additional live birth has been recently achieved by ICSI using fresh sperm [19].

Nevertheless, the use of the Hotchkiss technique to retrieve fresh sperm on the day of ART requires a trained staff in close proximity to the ART laboratory, and this procedure can be quite time consuming. This may lead to difficulties in using this technique to treat couples with RE. We previously developed and evaluated a new protocol, including a novel modified Hotchkiss technique in association with sperm cryopreservation, that facilitates the administration of ART to couples with RE [10].

The aim of this study was to describe this modified Hotchkiss technique and to present the pregnancy rate and birth rate achieved by ICSI with cryopreserved sperm retrieved from the bladder after refractory RE.

## Methods

### Patients

In this descriptive retrospective, single-center study, we analyzed the local database of all patients who banked sperm at the CECOS Laboratory Biology of Reproduction of La Conception University Hospital, Marseille, France, between 2004 and 2014.

We only included patients with RE who underwent urinary sperm retrieval by our modified Hotchkiss technique. RE was diagnosed by hypospermia (less than 2 ml in volume) associated with the presence of spermatozoa in the urine. For each patient who used his banked sperm for ART treatment, we collected information regarding age, aetiology of RE, ART technique used, number of cycles, fertilization rate, numbers of obtained embryos, transferred embryos, and frozen embryos, number of clinical pregnancies (i.e., heartbeat assessed by ultrasonography) and live birth rate.

### Modified Hotchkiss technique

Our modified Hotchkiss technique consisted of instillating 40 ml of a sterile culture medium for gametes (Ferticult®, FertiPro®, Beernem, Belgium) into the bladder at room temperature via aseptic catheterization. Water restriction was recommended for four hours before sperm retrieval. Ejaculation was achieved either by masturbation or by penile vibratory stimulation (PVS) (Ferticare®, Orion Medical, Silverado, California, USA) with midodrine adjunction in spinal cord-injured (SCI) patients [20, 21]. After ejaculation, ejaculate mixed with Ferticult® was collected by a new aseptic catheterization from the SCI patients or by voiding from the other patients. Samples were immediately centrifuged for 10 min at 1500 rotations per minute (420xg), and supernatants were removed. The pellets of spermatozoa were resuspended in Ferticult® and analysed as recommended by the WHO [22, 23].

### Sperm cryopreservation and post-thaw evaluation

The recovered spermatozoa were cryopreserved by progressive dilution in a cryoprotectant containing Glycerol (SpermFreeze®, FertiPro®, JCD, La Mulatière, France), according to the manufacturer’s instructions, introduced into high-security straws (CryoBioSystem®, L’Aigle, France), placed for 10 min in liquid nitrogen vapor, then plunged into liquid nitrogen [24].

A post-thaw evaluation was systematically performed as follows: one straw was thawed at room temperature for 10 min, Ferticult® (37 °C) was progressively added, and then the preparation was centrifuged for 10 min at 1500 rotations per minute (420xg) to separate the sperm from the cryoprotectant medium. The supernatant was discarded, and the pellet was divided into 2 μl droplets of Ferticult® on a Petri dish and covered with oil (IrvineScientific®, New town mount kennedy, Ireland). This preparation was placed at 37 °C for 10 min, and analyzed by inverted microscopy (Wilovert, Wild, Leica®, Nanterre, France) to assess total and progressive sperm mobility. Post-thaw evaluation was considered positive if motile spermatozoa (progressive or not) at the edge of each drop (and thus usable for ICSI) were observed. If no motile sperm were observed, 2 μl of pentoxifylline solution (Torental® 20 mg/ml, Sanofi Aventis, Paris, France) (final dilution 1/20 in the sperm suspension) was added to the droplet, and the preparation was re-analyzed after 20 min of incubation at 37 °C.

## Results

A total of 2171 patients banked sperm at the CECOS Laboratory Biology of Reproduction of La Conception University Hospital, Marseille, France between January 2004 and August 2014. A total of 3611 sperm cryopreservations were achieved.

**Fig. 1:**
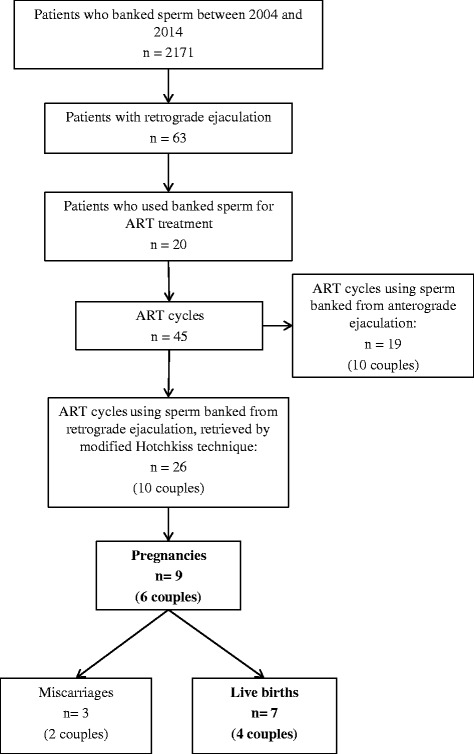
Flow-chart summarizing the characteristics and the ART results of patients included. ART: Assisted Reproduction Technique

Figure 1 summarizes the data on the included patients.

Ten RE patients used their banked straws for ART (mean number of cycles per couple: 2.6), the mean age at first sampling was 37 years (30–54), eight patients had a spinal cord injury, one a diabetic neuropathy, and one had a history of prostate surgery as an etiology of retrograde ejaculation.

The average total number of spermatozoa retrieved from the bladder was 8.7 million (0.1–20) and the average number of straws was 6.7 per couple (4–12).

Sperm parameters before and after thawing are shown in Table 1.

**Table 1:** Patient characteristics, sperm and cryopreservation parameters

Patient	Age	Etiology of RE	Initial parameters in bladder sperm retrieval			Number of straws	Sperm parameters at post-thaw evaluation after Pentoxifylline addition	
			Total number of spz retrieved from the bladder (Million)	Motile spermatozoa (%)	Spermatozoa with forward progression (%)		Motile spermatozoa/drop	Spermatozoa with forward progression (%)
1	33	SCI	20	10	5	5	5-20	5-10
2	30	SCI	12,2	10	5	8	5-20	0
3	30	SCI	3,5	2	2	12	5-20	20
4	34	SCI	11,8	25	5	6	>100	30
5	48	SCI	1	10	0	4	No	No
6	42	SCI	15,6	15	10	5	>100	30
7	54	TURP	0,1	60	10	8	20-100	20
8	39	SCI	0,5	10	5	7	5-20	5
9	31	D	2	30	10	6	20-100	60
10	37	SCI	1	5	0	6	<5	0

*RE* retrograde ejaculation, *SCI* Spinal cord injury, *TURP* transurethral resection of prostate, *D* Diabetes, *Spz* spermatozoa, *No* No post-thaw evaluation performed

All couples received ART treatment of in vitro fertilization (IVF) with ICSI. The results of ICSI cycles using frozen-thawed sperm retrieved by the modified Hotchkiss technique are shown in Table 2.

**Table 2:** Results of ICSI cycles using frozen-thawed sperm retrieved by Hotchkiss modified technique

Patients	Number of ICSI cycles performed	Fertilization rate (nb of 2PN zygotes/nb of mature oocytes) for each cycle	Number of embryos obtained for each cycle	Number (and quality*) of embryos transferred for each cycle	Number of frozen embryos for each cycle	Clinical pregnancies for each cycle	Live births (sex) for each cycle	Number of thawed embryos transferred for each cycle	Pregnancies after transfer of thawed embryos for each cycle	Live births after thawed embryo transfer
1	4	3/5; 5/5; 0/2; 2/4	3; 5; 0; 2	1(A); 2(blasto); 0; 2(blasto)	2; 0; 0; 0	0; 0; 0; 1	0; 0; 0; 2 (F,M)	2	0	0
2	2	2/6; 1/2	2; 1	2(B,B); 1(A)	0; 0	0; 0	0; 0	-	-	-
3	4	9/9; 9/9; 9/9; 3/5	9; 9; 9; 3	1(blasto); 1(blasto); 1(blasto); 1(B)	2;0;2;0	0; 1; 0; 0	0; 1 (F); 0; 0	-	-	-
4	1	2/3	2	1(B)	0	0	0	-	-	-
5	1	5/5	5	2 (A,B)	0	0	0	-	-	-
6	1	3/4	3	2(B,B)	0	1	1(F)	-	-	-
7	3	7/11; 1/1; 7/9	7; 1; 7	1(A); 1(A); 2(A,A)	2; 0; 1	0; 1; 0	0; 0; 0	2; 0; 1	0; 0; 1	0
8	5	0/4; 2/7; 5/14; 6/9; 1/10	0; 2; 5; 2; 1	0; 2(A,C); 2(B,B); 2(B,B); 1(B)	0; 3; 0; 0; 0	0; 0; 0; 0; 0	0; 0; 0; 0; 0	-	-	-
9	3	4/4; 6/9; 4/8	4; 6; 4	2(A,B); 2(A,B); 2(B,B)	1; 0; 0	1; 1; 1	0; 1(M); 1(M)	-	-	-
10	1	4/4	4	1	2	1	1(M)	-	-	-

*F* Female, *M* Male, *Blasto* blastocyst, *A* grade A embryo, *B* grade B embryo, *C* grade C embryo

*: embryo grade is expressed according to the ASEBIR embryo assessment criteria [30]

The average fertilization rate was 62.6 % per cycle (0–100).

Of the 26 cycles, nine clinical pregnancies were achieved (34.6 % clinical pregnancies per cycle and 37.5 % clinical pregnancies per transfer), including eight after fresh embryo transfer and one after thawed embryo transfer, resulting in seven live births. The average live birth rate per transfer was 28 %.

In total, of the ten couples treated with the modified Hotchkiss technique, 6 (60 %) obtained at least one clinical pregnancy and 4 (40 %) achieved at least one live birth.

## Discussion

To our knowledge, this is the largest series of births achieved using spermatozoa retrieved from post-ejaculatory urine by the new MHT. Our novel protocol is based on an asynchronous management, involving systematic freezing of spermatozoa recovered from the bladder.

Despite the length of time since the first description of the Hotchkiss technique [9] and the publication of many variants, a limited number of births among couples with a male suffering from RE has been reported using this technique [4]. This low number could be explained by the following:

- The majority of patients with RE achieved antegrade ejaculation (total or partial) after medical treatment [4], therefore the antegrade ejaculate was used for ART treatment. In our series, 10 of the 20 RE patients with banked sperm achieved antegrade ejaculation.
- Reluctance to perform the technique because of the risks associated with bladder catheterization, such as stricture or infection, especially in diabetic patients, or increased discomfort [25, 26]. Nevertheless, this hypothesis is not relevant concerning SCI patients. Indeed, most of them regularly use aseptic catheterization to empty their bladder.
- For spinal cord injured patients, the use of PVS with midodrine adjunction lengthens the time required for sperm retrieval compared to the time required by other patients, such as those with diabetes or idiopathic RE, and requires close patient monitoring because of the increased risk of autonomic dysreflexia [20, 21].

Thus, instead of the Hotchkiss technique, oral alkalinization of urine is performed by some clinicians. Attempts to manipulate physicochemical qualities of urine via fluid intake require ingestion of a large amount of liquid on a specified schedule during the hours preceding the retrieval [8, 27]. These protocols are used by numerous centers that treat RE patients because they do not require special equipment or surgical procedures.

However, changes in osmolarity occur rapidly under these conditions, placing the patient at risk for failure if the procedure is disrupted [8, 10].

Our modification of the Hotchkiss’ technique involves the use of a culture medium for gametes and embryos (Ferticult®). We previously compared the characteristics of sperm obtained from the bladder after oral alkalinization of urine versus that acquired from endovesical instillation of Ferticult® in the same patients and showed that endovesical instillation statistically improved sperm characteristics [10]. Other adaptations of this technique that resulted in successful live births via IUI or ICSI employed basic cell culture medium (Earle’s balanced salt solution buffered with HEPES [14], basal medium BM-1 [19], a low-electrolyte solution (glucose) [17]), or a culture medium used for human embryo or gamete culturing (human tubal fluid with 5 % BSA) [18]. The basic cell culture media are less expensive and have yielded successful births, but the specific culture medium for gametes is perfectly suited for use with this technique.

Our novel protocol also includes a systematic cryopreservation of the retrieved sperm. The use of cryopreserved spermatozoa does not affect the fertilization rate achieved with ICSI and greatly facilitates medical management [28, 29]. Indeed, achieving aseptic bladder catheterization on the day of ART is time consuming and is not possible in all ART centers because it requires the completion of an aseptic catheterization in close proximity to the laboratory. In our series, an average of 6.7 straws were frozen for each patient and the post-thaw evaluations were always positive. Prior post-thaw evaluation allows for validation of straw quality in advance. Finally, this new technique minimises the risk of repeated catheterizations, allowing the female partner to avoid the constraints of stimulation in the case of impossible or insufficient sperm retrieval on the day of oocyte retrieval. However, we acknowledge this technique has an implied use in IVF, which is more invasive for the female partner than IUI treatment.

## Conclusions

This study demonstrated that the new MHT allowed for successful sperm cryopreservation, leading to the efficient and easier management of couples with refractory RE. The collection of fresh spermatozoa from the male partners is simple and easy at trained fertility centres. Although it is completely possible to achieve live births by a series of IUIs with fresh sperm, our protocol allows for the simple asynchronous management of these couples immediately following the banking of a sperm sample.

## Abbreviations

ART: Assisted reproductive technology
CECOS: Centre for the Study and Preservation of Eggs and Sperm (Centre d’Etude et de Conservation des Œufs et du Sperme)
ICSI: Intra cytoplasmic sperm injection
IUI: Intrauterine insemination
IVF: In vitro fertilization
MHT: Modified Hotchkiss technique
PVS: Penile vibratory stimulation
RE: Retrograde ejaculation
SCI: Spinal cord-injured
